# Dipyridamole ameliorates doxorubicin-induced cardiotoxicity

**DOI:** 10.25122/jml-2021-0199

**Published:** 2022-09

**Authors:** Esraa Alyasiry, Ali Janabi, Najah Hadi

**Affiliations:** 1 Department of Pharmacology and Toxicology, College of Medicine, Jabir Ibn Hayyan Medical University, Najaf, Iraq; 2 Department of Pharmacology and Toxicology, Faculty of Pharmacy, University of Kufa, Najaf, Iraq; 3 Department of Pharmacology and Therapeutics, Faculty of Medicine, University of Kufa, Kufa, Iraq

**Keywords:** dipyridamole, doxorubicin-induced cardiotoxicity, inflammatory mediators, oxidative stress, apoptotic factors

## Abstract

Dipyridamole is a platelet inhibitor with antithrombotic properties that can help prevent stroke recurrence. Twenty-eight male rats were divided randomly into four groups (7 rats in each group). Control group: rats received a natural diet and water. Normal saline group: rats received 0.9% normal saline for two weeks. Doxorubicin group (induced group): rats received 2.5 mg/kg three times a week for two weeks. Dipyridamole group (dipyridamole treated group): received dipyridamole (6 mg/kg/daily) orally for two weeks. Doxorubicin caused cardiotoxicity as indicated by a significant increase in tumor necrosis factor-α, interleukin-6, malondialdehyde, and caspase-3 level (P<0.05), while total antioxidant capacity and Bcl-2 levels were significantly reduced in cardiac tissues of rats in the doxorubicin group compared to the normal saline control group (P<0.05). Dipyridamole significantly ameliorates doxorubicin-induced cardiotoxicity, as suggested by a significant decrease in inflammatory markers (tumor necrosis factor-α and interleukin-6) (P<0.05). Moreover, the cardiac tissue level of oxidative marker malondialdehyde was significantly decreased (P<0.05), and total antioxidant capacity significantly increased in the dipyridamole group in comparison to the doxorubicin-only group (P<0.05). Dipyridamole exerted a significant heart-protective effect against doxorubicin-induced cardiotoxicity in rats, probably via interfering with oxidative stress, inflammatory response, and apoptotic pathway. The goal of this study was to investigate the potential protective effect of dipyridamole against doxorubicin-induced cardiotoxicity via interfering with pro-inflammatory, oxidative, and apoptotic pathways.

## INTRODUCTION

Cardiotoxicity is a toxicity that impacts the heart, indicated by heart electrophysiology dysfunction and/or muscle injury. During cardiotoxicity, the heart is weakened and loses the capacity to pump blood efficiently. It also involves hemodynamic flow alterations or thrombotic events, not only direct effects on the heart due to cancer treatment [1]. Cardiotoxicity can occur early or later in the disease, and symptoms can range from mild myocardial dysfunction to permanent heart failure or death [2]. The possible mechanisms of chemotherapy-induced cardiotoxicity include:

1. Coagulation-systems effects (ischemic events, thrombogenesis, and vascular toxicity);
2. Direct cellular toxicity (progressive myocardial damage and diastolic and systolic dysfunction);
3. Arrhythmogenic effects;
4. Hypertensive effects;
5. Inflammation of the myocardium and/or pericardium in the form of pericardial disorders or myocardial failure [3, 4].

Doxorubicin (DOX) is an anthracyclines anticancer drug with a broad spectrum of activity effective against hematological and solid tumor cancers. The therapeutic function of DOX is accomplished by intercalating into DNA, inhibiting topoisomerase II, and shutting down DNA and RNA production [5]. In the early 1960s, doxorubicin and daunorubicin were discovered in the pigment bacterium Streptomyces paucities [6]. Doxorubicin and other anthracyclines have long been suspected of causing cardiotoxicity by mechanisms unrelated to their antitumor activity. This has given rise to expectations that treatment protocols can protect the heart while not reducing the antitumor activity of the drug [6]. However, the actual mechanism through which doxorubicin causes cardiotoxicity is uncertain. Increased lipid peroxidation, oxidative stress, endoplasmic reticulum mediated apoptosis, DNA/RNA damage, calcium homeostasis disruption, and autophagy suppression appear to be multifactorial mechanisms causing DOX-induced cardiotoxicity [7–9].

Dipyridamole (DP) is a platelet inhibitor with antithrombotic properties that can help prevent stroke recurrence. DP was first used as a cardioprotective coronary vasodilator in the early 1960s. DP has antiplatelet and vasodilatation properties; the action mechanism is likely linked to platelet phosphodiesterase inhibition, prostacyclin release stimulation, or adenosine uptake inhibition. It has additional pleiotropic benefits, such as anti-inflammatory and antioxidant properties, in addition to its function as a platelet inhibitor [10, 11]. DP has a direct anti-inflammatory effect via inhibition of platelet-monocyte interaction and an indirect anti-inflammatory effect via PGI2 and adenosine. Monocytes secrete monocyte chemotactic protein-1 (MCP-1) and matrix metalloproteinase 9 (MMP-9) due to activated platelets adhering to and stimulating them. When activated platelets are treated with DP, they become more active. DP has antioxidant properties because it inhibits reactive oxygen radicals (ROS) in endothelial cells and platelet. The antioxidant effect of DP in vascular cells has been partly mediated by suppressions of inflammatory nuclear factor-kB (NF-kB) signaling. Dipyridamole pleiotropic antioxidant properties can help stabilize vascular membrane and platelet and prevent low-density lipoprotein oxidation, which could explain some of the drug's therapeutic benefits [12, 13]. Dipyridamole improves the cGMP-dependent downstream vasodilatation effect in the smooth muscle by inhibiting cGMP phosphodiesterase (PDE). DP may also stimulate prostacyclin (PGI2) development by increasing intracellular cAMP levels. PGI2 is a potent platelet aggregation inhibitor vasodilator. In cells, including endothelial cells, a cyclooxygenase-dependent pathway produces PGI2 by raising local adenosine levels, and thus, dipyridamole enhances vasodilatation. Thus, dipyridamole may also have an indirect vasodilatation effect on the vascular smooth muscles [14].

## MATERIAL AND METHODS

### Animal preparation

Overall, 28 male Sprague Dawley rats were obtained from the University of Kufa/Faculty of Science, weighing 200–300 g and aged 10–11 weeks. The rats were kept at the University of Kufa/Faculty of Pharmacy animal house. The animals were kept in a separate chamber, in a group caging system, with temperature and humidity regulated at 24±2℃. Water and a standard chow diet were given ad libitum in their cages. The animals were left for two weeks to reduce the stress caused by the change in their environment. All experimental procedures were conducted following the principles of care and use of laboratory animals in research.

### Study design

A total of 28 male rats were split randomly into four groups (7 rats per group): control group: rats received a natural diet and water during the whole study period; normal saline (N/S) group: rats received a 0.9% normal saline dose for two weeks, where N/S is the vehicle of dipyridamole and is considered a control; doxorubicin (DOX) group (induced group): rats received 2.5 mg/kg three times a week for two weeks by intraperitoneal (IP) injection (a cumulative dose of 15 mg/kg) [15]. Dipyridamole group (treated group): rats received a dose of 6 mg/kg/daily orally over two weeks [16], and DOX was also given in the same way as in the DOX group.

### Preparation of the drugs

Dipyridamole powder was acquired from the Sigma- Chemical Company in the U.S.A. DP was dissolved in 0.9% N/S (2 mg/ml as stock solution) [17]. Individual doses were given according to 6 mg/kg/day.

### Collection of Samples

#### Blood sample collection

Each animal's body weight was measured 48 hours before the last dose of DOX. We used ketamine at a dose of 100 mg/kg and xylazine at 10 mg/kg to anesthetize the animals. First, animals were immobilized, a thoracoabdominal incision was made, and blood was drawn immediately from the heart's left ventricle through a heart puncture. Then the serum was collected by centrifugation at 5,000 rpm for 10 minutes. After that, blood samples were placed in tubes with the clot activator gel. The serum obtained was used to evaluate cTnI, IL-6, and TNF-α using the ELISA technique.

#### Tissue sample preparation

The hearts were taken out, cleaned, and weighed before being sliced into apical and basal sections. Clots were removed by rinsing the basal side of the heart with ice-cold saline and then stored in a deep freeze (-80 C). Each part was weighed after melting and homogenized using an ultrasonic liquid processor with high intensity in 1:10 (w/v) 0.1-M phosphate-buffered saline (pH-7.4) containing 1% triton x-100 and protease-inhibitors cocktails [18]. The homogenate was centrifuged at 5,000 rpm at 4℃ for 10 minutes. The supernatants were utilized to evaluate malondialdehyde (MDA) level, caspase-3, and Bcl-2 using available ELISA kits and total antioxidant capacity (TAC) using colorimetric assay kits as directed by the manufacturer guidelines (Elabscience, U.S.A.).

#### Tissue Sampling for Histopathology

For histopathological exams, the apical portion was saved and fixed in 10% neutral formalin, then embedded in paraffin block and sliced into parts with a thickness of 5 µM using a microtome. Light microscopy was used to investigate tissue sections stained with hematoxylin and eosin (H&E) [19].

### Measurement of study parameter

The levels of IL-6, TNF-α, Casp-3, and Bcl-2 were determined using an ELISA kit obtained from Melsin, China, according to the manufacturer's instructions. The level of MDA was determined utilizing the corresponding ELISA kits obtained from Elabscience, U.S.A., according to the manufacturer's instructions.

### Statistical-Analysis

Analyses were performed using SPSS version 26. Data were expressed as mean+standard error means (SEM). We used one-way ANOVA for the comparisons among all groups, followed by post hoc testing using the LSD method. Mann-Whitney, Kruskal-Wallis and one-way ANOVA were also utilized to compare histopathology changes in different groups. In all tests, the statistically significant level was set at P<0.05.

## RESULTS

**Figure 1:**
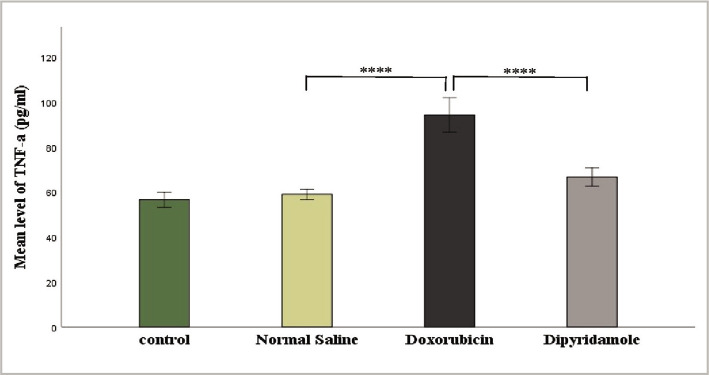
Serum TNF-α level of experimental groups.

**Figure 2:**
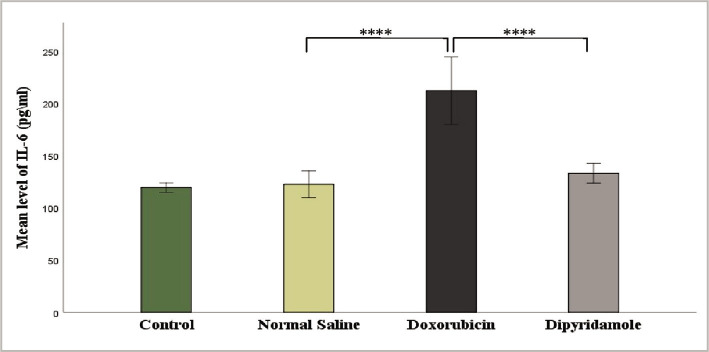
Serum IL-6 level of experimental groups.

**Figure 3:**
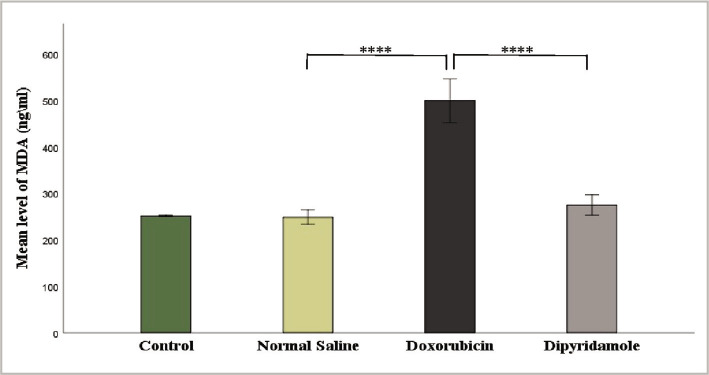
MDA level of experimental groups.

**Figure 4:**
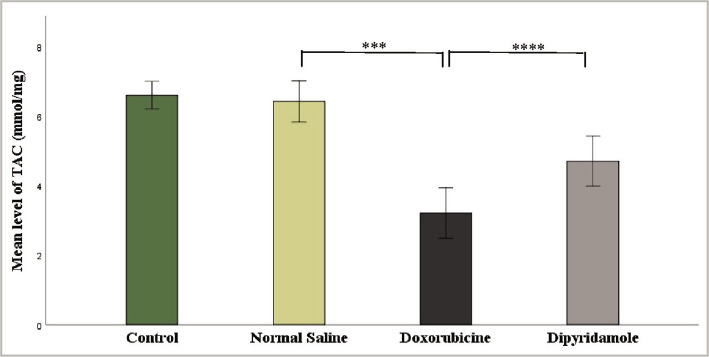
TAC level of experimental groups.

**Figure 5:**
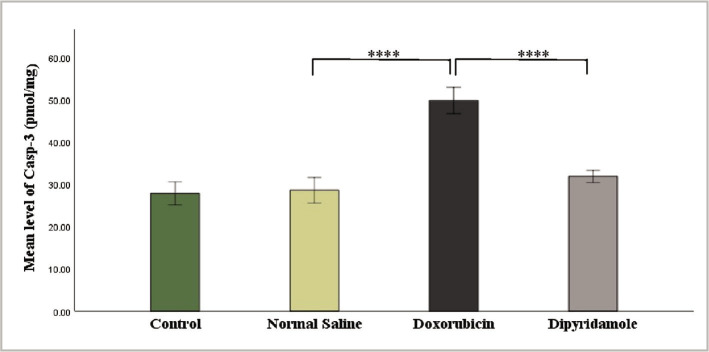
Caspase-3 level of experimental groups.

**Figure 6:**
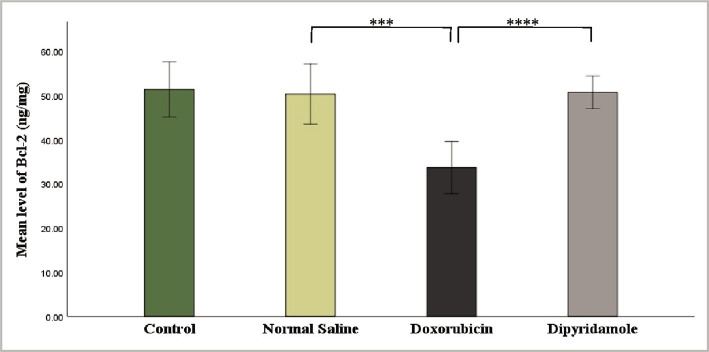
Bcl-2 level of experimental groups.

The injection of 2.5 mg/kg of doxorubicin caused cardiotoxicity as indicated by a significant increase in TNF-α, IL-6, MDA, and caspase-3 levels, while TAC and Bcl-2 levels were significantly reduced in cardiac tissues of rats in the doxorubicin group when compared to N/S group. Dipyridamole significantly ameliorated doxorubicin-induced cardiotoxicity, as suggested by the significantly decreased inflammatory mediators TNF-α and IL-6 (Figures 1 and 2). Cardiac tissue level of the oxidative marker MDA significantly decreased (Figure 3) and also significantly increased in TAC (Figure 4) with the dipyridamole group compared to the doxorubicin-only group. Additionally, dipyridamole significantly attenuated doxorubicin-induced apoptosis, reflected by lower cardiac caspase-3 level (Figure 5) and significant elevation in Bcl-2 compared to the doxorubicin-only group (Figure 6). Furthermore, DP significantly improved the cardiomyopathy histological lesions scores compared to the doxorubicin group.

Rats were treated with either vehicle (N/S), DOX (2.5 mg/kg), DOX (2.5 mg/kg)+DP (6 mg/kg) or untreated (control). Serum TNF-α concentration was determined using a TNF-α ELISA kit. Data are mean±SEM. ****P<0.0001 *vs*. N/S group by post-hoc using LSD multiple comparison tests following one-way ANOVA.

Rats were treated with either vehicle (N/S), DOX (2.5 mg/kg), DOX (2.5 mg/kg)+DP (6 mg/kg) or untreated (control). Serum IL-6 concentration was determined using an IL-6 ELISA kit. Data are mean±SEM. ****P<0.0001 *vs*. N/S group by post-hoc using LSD multiple comparison test following one-way ANOVA.

Rats were treated with either vehicle (N/S), DOX (2.5 mg/kg), DOX (2.5 mg/kg)+DP (6 mg/kg) or untreated (control). The cardiac MDA concentration was determined using the MDA ELISA kit. Data are mean±SEM. ****P<0.0001 *vs*. N/S group by post-hoc using LSD multiple comparison test following one-way ANOVA.

Rats were treated with either vehicle (N/S), DOX (2.5 mg/kg), DOX (2.5 mg/kg) + DP (6 mg/kg) or untreated (control). The cardiac TAC concentration was determined using the TAC ELISA kit. Data are mean±SEM***P<0.001, ****P<0.0001 *vs*. N/S group by post-hoc using LSD multiple comparison test following one-way ANOVA.

Rats were treated with either vehicle (N/S), DOX (2.5 mg/kg), DOX (2.5 mg/kg)+DP (6 mg/kg) or untreated (control). The cardiac casp-3 concentration was determined using a casp-3 ELISA kit. Data are mean±SEM. ****P<0.0001 *vs*. N/S group by post-hoc using LSD multiple comparison tests following one-way ANOVA.

**Figure 7:**
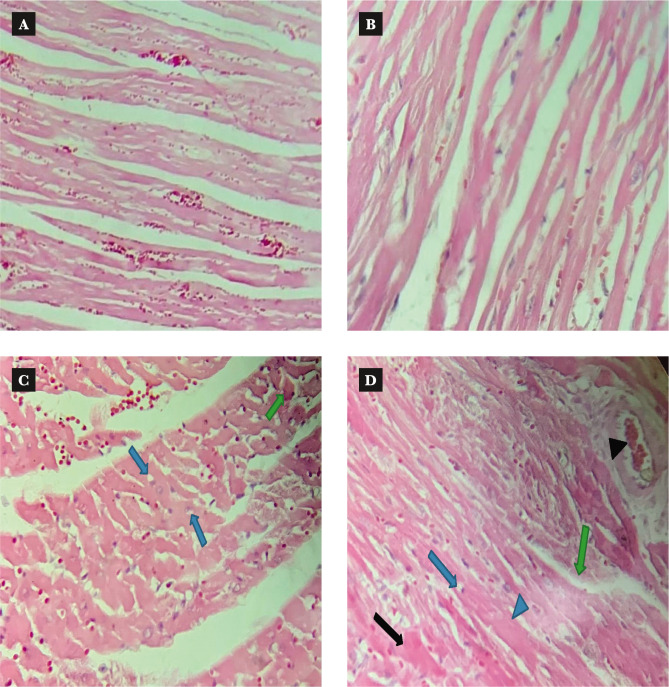
A – The myocardium of the control group: the appearance has zero damage showing normal histology H&E. B – the myocardium of vehicle (N/S) group: the appearance has zero damage showing normal histology H&E. C – the myocardium of DOX-treated in the rat showed severe histopathological injury distinguished by cellular swelling (green arrow), and perinuclear vacuolation and cytoplasm vacuolization (blue arrow) H & E. D – the myocardium of doxorubicin (2.5 mg/kg) plus dipyridamole-treated (5 mg/kg) rats demonstrated a mild to moderate histopathological injury distinguished cellular swelling (black arrowhead), interstitial edema (green arrow), perinuclear vacuole (blue arrow), congestion (black arrowhead) and some karyolysis necrosis cells (black arrow), H&E.

Rats were treated with either vehicle (N/S), DOX (2.5 mg/kg), DOX (2.5 mg/kg)+DP (6 mg/kg) or untreated (control). The cardiac BCL-2 concentration was determined using the BCL-2 ELISA kit. Data are mean±SEM. ***P<0.001, ****P<0.0001 *vs*. N/S group by post-hoc using LSD multiple comparison test following one-way ANOVA (Table 1, Figure 7 A–D).

**Table 1:** Mean of histopathological score and comparison among experimental groups.

Group	SEM±Mean	Comparison	P-Value
Control	.00±0.000	Control vs. Normal Saline	1.000
Normal Saline	.00±0.000	Normal Saline vs. Dipyridamole	0.001
Doxorubicin	3.71±0.184	Doxorubicin vs. Normal Saline	0.001
Dipyridamole	2.43±0.297	Doxorubicin vs. Dipyridamole	0.007

## DISCUSSION

In this study, dipyridamole significantly reduced the elevation of pro-inflammatory markers, IL-6, and TNF-α in rats (Figures 1 and 2) compared to the induced untreated DOX group suggesting that dipyridamole has a protective effect on cardiotoxicity induced by DOX. DP significantly decreased the plasma TNF-α and IL-6 levels compared with the induced untreated DOX group in the ischemic brain. Its neutralization reduces cerebral ischemic damage in a rat model [20, 21]. A study showed that dipyridamole, when given before resuscitation after hemorrhagic shock in rats, protected the myocardium against dysfunction and injury by reducing the inflammatory response to shock and resuscitation [22]. Furthermore, in a rat model, Elsherbiny demonstrated that dipyridamole significantly decreases TNF-α and IL-6 levels in diabetic renal injury compared to the induced untreated DOX group [16].

In another animal study, the cardioprotective impact of antiplatelet medications, particularly cilostazol, was also demonstrated, with Dox-induced elevation of these pro-inflammatory cytokines (TNF-a, IL-6) greatly reduced. These side effects could be linked to their phosphodiesterase inhibitor [23]. These findings agree with those obtained by Mete et al., who suggested that cilostazol inhibited the production of cytokines, especially TNF- α, possibly related to their ability to raise cAMP [24]. The pivotal role of oxidative stress in DOX-induced cardiotoxicity is well confirmed and documented. Therefore, one of the objectives of the present study was to evaluate the antioxidant potential of DP in DOX-treated rats. The study showed that DP significantly decreased lipid peroxidation and preserved the antioxidant status of the heart tissue as indicated in reduction of MDA level (Figure 3) and elevation of TAC level (Figure 4) in DP plus DOX treated rat compared to DOX only treated rat. To our knowledge, this is the first study designed to evaluate the effect of DP on DOX-induced cardiotoxicity. However, the antiplatelet drug cilostazol, in the previous study, showed that Dox caused a significant increase in MDA levels (oxidant system) and a decrease in TAC activity (antioxidant enzyme) when compared to the control group [23]. MDA levels are considered an indicator of excessive free radical generation largely arising from oxidative products of DOX, which can harm the heart. These findings suggested that cilostazol could protect against DOX-induced oxidative stress by scavenging free radicals and/or boosting the activity of endogenous antioxidants. Dipyridamole antioxidant properties may help protect against testicular chemical/reperfusion (I/R) injury. Vargas and colleagues discovered that dipyridamole probably scavenges reactive oxygen radicals generated by polymorphonuclear leukocytes in investigating its antioxidant effects [25]. Another animal study reported that dipyridamole protects liver cells against warm I/R injury [26].

In addition, the neuroprotective effects of dipyridamole were discovered when utilized to protect the brain against I/R injury in a rat model of cerebral ischemia [20, 21]. In this study, dipyridamole inhibited the last steps of cell apoptosis, as reflected by a considerable decrease in the level of caspase-3 (Figure 5) and an increase in the level of Bcl-2 (Figure 6) in cardiac tissue compared to the DOX group. To our knowledge, this is the first study designed to examine the effect of dipyridamole on apoptosis in DOX-induced cardiotoxicity. However, a previous study revealed that diabetes increased caspase-3 in diabetic rat kidneys, while the dipyridamole treatment reduced the activities of caspase-3 in diabetic rat nephropathy [16]. In addition, dipyridamole was previously reported to suppress the activities of caspase-3 in models of hepatocellular carcinoma [27]. The histopathological finding of the present study revealed the ability of DP to attenuate the lesions of cardiac tissue induced by DOX as indicated by improvement in cardiomyopathy severity score to a notable extent compared to DOX only group. Treatment of rats with the antiplatelet drug, namely cilostazol, showed less cardiac histological alteration when compared with DOX treated rats. In addition, there was a marked reduction in the severity of the muscle injury, moderate vacuolization of the cytoplasm, and minor inflammation [28]. Data in this study demonstrated that DP interfered with oxidative pathway, as indicated by lowering lipid peroxidation and preserved cardiac antioxidant status and suppressed the inflammatory response as indicated by reducing TNF-α and IL-6 as well as inhibiting the terminal step of apoptosis reflected by decreased caspase-3 level and increased Bcl-2 level in cardiac tissue. All these data might provide mechanistic answers for the cardio-protective effect of DP in DOX-induced cardiotoxicity.

## CONCLUSIONS

Based on the current results, we can conclude that DP effectively reduces DOX-induced cardiotoxicity in rats. DP significantly lowers the oxidative stress, inflammatory response, and apoptosis induced by DOX in rat serum and cardiac tissue, which may be a reasonable explanation for their cardioprotective effect.
